# Association between social participation and hope levels in stroke patients: a cross-sectional study

**DOI:** 10.3389/fpsyg.2025.1674893

**Published:** 2025-10-29

**Authors:** Miao Liu, Yan Liu, Tian Li, Jiying Yang, Juan Wang, Hen Wang

**Affiliations:** Renmin Hospital of Wuhan University, Wuhan, China

**Keywords:** stroke, rehabilitation, social participation, hope, correlational study

## Abstract

**Objectives:**

Exploring Factors Influencing the Level of Hope in Stroke Patients and Its Relationship with Social Participation.

**Methods:**

We conducted a cross-sectional study at Wuhan University People’s Hospital from March to June 2024 and performed a correlation analysis on the sample data. Using convenience sampling, we enrolled 122 stroke patients who met the inclusion criteria. Participants completed questionnaires assessing social participation (Impact on Participation and Autonomy Questionnaire, IPA), hope (Herth Hope Index, HHI), activities of daily living (Barthel Index), and sociodemographic/disease characteristics. Data were analyzed using SPSS 26.0 with descriptive statistics, *t*-tests, ANOVA, Pearson correlation, and multiple linear regression. All statistical tests were two-tailed with significance set at *p* < 0.05. Effect sizes were reported with 95% confidence intervals.

**Results:**

Participants demonstrated moderate social participation impairment (IPA: 32.60 ± 16.32), with the most pronounced limitations in autonomous participation in family roles (subscore: 11.10 ± 6.17). Hope levels averaged within the moderate range (HHI: 30.81 ± 7.28). Furthermore, significant negative correlations existed between all dimensions of social participation and all dimensions of hope (*p* < 0.01). Regression analysis indicated that Barthel Index and social participation were key factors influencing hope levels among stroke patients.

**Conclusion:**

This study demonstrates that stroke survivors exhibit moderate impairments in social participation and hope levels. These findings suggest that rehabilitation programs should prioritize social participation enhancement while providing targeted interventions for female patients and those with lower socioeconomic status or poorer functional ability. Further longitudinal research is needed to establish causal relationships and optimize intervention strategies.

## 1 Introduction

Stroke is the second leading cause of death in the world and the third leading cause of death and disability combined. The global cost of stroke is estimated to exceed $721 billion (0.66% of global GDP) (Nandan et al., 2023). Acute stroke patients often suffer from varying degrees of psychological disorders, neurological deficits, and limitations in performing daily living tasks, and most survivors retain varying degrees of disability despite effective rehabilitation and treatment (Wu et al., 2019), with participation dysfunction being the most prominent sequela (Cieza and Kostansjek, 2021). Social participation, an important outcome indicator of disease recovery (Pokryszko-Dragan et al., 2020), refers to participation in life situations, which is the behavioral process and subjective experience of an individual who actively participates in family or social activities and integrates into the family or social environment (Qu et al., 2024). Limited social participation can negatively affect patients’ mental health and life satisfaction, among others (Ge et al., 1844). Studies have shown (Masuccio et al., 2024) that the level of social participation can contribute to emotional regulation, enhance rehabilitation potential, and promote stroke recovery. Hope is a potential psychological force that influences people’s behavior and the way they deal with things, and different levels of hope can reflect different attitudes toward the disease (Western, 2007). Hopeful beliefs can make stroke patients feel confident and courageous, helping them adapt to and overcome difficulties (Ni et al., 2021). The higher the level of hope, the more positive the patients’ attitude toward the disease. The opposite is true for patients with low levels of hope. Systematic reviews (Kruithof et al., 2013; Northcott et al., 2016) have found associations between poor social support and depression, reduced quality of life, and worse physical recovery among stroke survivors. Perceived social support shows significant temporal associations with greater community participation (Erler et al., 2019), lower depression levels (Volz et al., 2016), and better functional status following stroke onset (Tsouna-Hadjis et al., 2000; Villain et al., 2017). Hope may be an essential coping strategy and a psychological resource for patients with chronic illness, including stroke survivors. Most relevant studies (Alaszewski and Wilkinson, 2015; Bright et al., 2020; Bright et al., 2011; Cross and Schneider, 2010; Levy et al., 2022; Soundy et al., 2014; Tutton et al., 2012; Visvanathan et al., 2019) have adopted a qualitative approach to investigate the relevance of hope in the perspectives and recovery experiences of stroke survivors. Additionally, impaired functional status increases patients’ emotional distress, feelings of hopelessness, and perceived stress while hindering social interactions and the rehabilitation process (Fong et al., 2022). Therefore, the degree of disability in stroke patients may influence their level of hope.

To our knowledge, no empirical studies have examined the relationship between social participation feeling and hope or the potential mediating role of hope in improving the functional outcomes of stroke survivors. This study aims to: (1) assess social participation and hope levels in stroke survivors, (2) examine their interrelationship, and (3) identify modifiable associated factor of hope to develop evidence-based nursing interventions for improving psychosocial outcomes.

## 2 Materials and methods

### 2.1 Participants

This cross-sectional study employed correlation analysis to examine the relationships between key variable. The study was approved by the Ethics Committee of Renmin Hospital of Wuhan University (approval number: WDRY2024-K065). The study was conducted in accordance with the Declaration of Helsinki. Stroke patients admitted to the People’s Hospital of Wuhan University from March and June 2024 were selected for the study using a convenience sampling method. Inclusion criteria: (1) those who had cerebral infarction or cerebral hemorrhage confirmed by CT or MRI and met the diagnostic criteria in the Chinese guidelines for the diagnosis and treatment of acute stroke (Liu et al., 2023); (2) aged ≥18 years (3) non-acute stage, currently in stable condition, clearly conscious, with normal speech comprehension; and (4) patients who gave informed consent to this study and participated voluntarily. Exclusion criteria: (1) suffering from mental illness or mild cognitive impairment, unable to cooperate with the study; (2) suffering from disabilities, malignant tumors, and other serious physical diseases or other cerebrovascular diseases such as Parkinson’s, epilepsy, etc.; (3) complaining of severely impaired vision and hearing.

The researchers explained the study’s purpose and significance to participants, emphasizing that their participation was anonymous, data would remain confidential, and results would be used solely for research purposes. After obtaining informed consent from the patient, the questionnaire was distributed, and the on-site survey was conducted in a one-on-one format. Patients completed the survey independently and objectively. If they encountered difficulties, the investigators assisted by reading the questions aloud and recording their responses. The questionnaires were collected on the spot, and a total of 130 questionnaires were distributed, of which eight questionnaires were excluded: 5 for the same answers for each question, and 3 for deviation from the inclusion criteria. And the valid recovery rate of the questionnaires was 93.84%.

### 2.2 Measures

#### 2.2.1 Demographic data

Sociodemographic and clinical characteristics of stroke patients were collected during rehabilitation, including gender, age, religion, marital status, residential area (urban/rural), education level, health insurance type, employment status, monthly household income per capita, stroke type (ischemic/hemorrhagic), first-onset status, and number of post-stroke sequelae (e.g., hemiplegia, speech impairment, sensory deficits, and dysphagia).

#### 2.2.2 Social participation

The Impact on Participation and Autonomy (IPA) questionnaire (He et al., 2025) was used for the survey. The scale consists of 25 items with four dimensions, namely, indoor autonomy (7 items), outdoor autonomy (5 items), family role autonomy (7 items), and social life autonomy (6 items). Each item was assigned a score from 0 to 4, ranging from “very likely to participate” to “very unlikely to participate,” with higher scores indicating poorer levels of social participation. Previous validation studies have established the scale’s strong reliability and validity. The most recent evaluation reported a Cronbach’s alpha coefficient of 0.962 (He et al., 2025), confirming its internal consistency. This instrument has been extensively utilized across various clinical research contexts. In this study, Cronbach’s coefffcient *α* for the IPA scale was 0.924, with the values of α for the dimensions ranging from 0.834 to 0.878.

#### 2.2.3 Hope

Hope was measured using the Herth Hope Index (HHI) (Snyder et al., 1991), which consists of 12 items and three dimensions: a positive attitude towards reality and the future, taking positive action, and maintaining close relationships with others. The Herth Hope Index (HHI) utilizes a 4-point Likert scale (1 = completely disagree to 4 = completely agree), with items 3 and 6 reverse-scored. Total scores are calculated by summing all items, where higher scores indicate greater levels of hope. All 12 items had a significant loading on one of the three factors as originally formed subscales of the HHS. The HHI has a high level of reliability with reported Cronbach’s alpha ranging from 0.74 to 0.84, and ranging from 0.77 to 0.79 in the present study (Dorsett et al., 2017). In this study, Cronbach’s *α* for the HHI scale was 0.82, and the Cronbach’s α for the dimensions were between 0.780 and 0.827.

#### 2.2.4 Barthel index

This scale is the most frequently used clinical method for assessing self-care ability. It focuses on seven self-management activities, including eating, dressing, personal hygiene, toileting, bathing, bowel control, and urination control. In addition, three mobility activities were focused on, namely bed and chair transfers, walking, and walking up and down stairs. Different items and levels are scored 0, 5, 10, and 15 out of a total of 100 points. Self-care scoring criteria: 60 or more points are considered basic self-care; 40–60 points are considered as needing help; <40 points are considered as basically needing full help. The Cronbach’s *α* coefficient for the Barthel Index is 0.860 (Cid-Ruzafa and Damián-Moreno, 1997). In this study, Cronbach’s coefffcient α for the BI was 0.885.

### 2.3 Data analysis

Statistical analyses were performed using SPSS 26.0 (IBM Corp, Armonk, NY). Categorical variables were presented as frequencies and percentages. We performed the Kolmogorov—Smirnov test to assess normality of distribution. Descriptive statistics characterized the subjects’ sociodemographic characteristics: categorical variables were presented as counts and percentages, while continuous variables were expressed as mean ± standard deviation (SD). Between-group comparisons were conducted using independent samples *t*-tests (two groups) or one-way ANOVA (multiple groups). Pearson correlation analysis examined relationships between hope levels and social participation. Multiple linear regression analysis assessed the influence of demographic characteristics and social participation on hope levels. A two-tailed *p*-value <0.05 was considered statistically significant.

## 3 Results

### 3.1 Stroke patients’ level of social participation and level of hope scores

The total social participation level of the 122 patients was (32.60 ± 16.32); the total hope level was (30.81 ± 7.28) (see Table 1).

**Table 1:** IPA and Herth Hope Scale scores in 122 stroke patients (points, x ± s).

Item	Score
Total IPA score	32.60 ± 16.32
Indoor autonomous participation	9.95 ± 5.92
Outdoor autonomous participation	3.47 ± 3.51
Autonomous participation in family roles	11.10 ± 6.17
Autonomous participation in social life	8.08 ± 5.91
Herth hope scale total score	30.81 ± 7.28
Taking positive action	10.31 ± 2.93
Positive attitude towards reality and future	10.46 ± 3.20
Maintaining close relationship with others	10.03 ± 3.15

### 3.2 Comparison of Herth Hope Scale scores of stroke patients with different general information

The results showed that the comparison of Herth Hope Scale scores of stroke patients with different genders, educational levels, family income levels, and Barthel indexes showed statistically significant differences (*p <* 0.01) (see Table 2).

**Table 2:** Comparison of Herth Hope Scale scores in 122 stroke patients with different general information (score, x ± s).

Item	Case	Herth Hope Scale score	*t/F*	*P*	Cohen’s d/η^2^
Gender					*0.462*
Male	69	32.14 ± 7.01	−2.53	0.013	
Female	53	28.85 ± 7.30			
Age					0.017
<50	23	31.74 ± 5.67	1.04	0.357	
50 ~ 70	69	31.73 ± 6.62			
>70	30	29.77 ± 6.84			
Educational level			4.65	0.01	0.072
Junior high school and below	40	28.97 ± 7.41			
High school and junior college	73	30.72 ± 6.89			
College and above	19	37.11 ± 7.10			
Marital status			0.387	0.7	0.88
Married	98	31.37 ± 6.57			
Other (unmarried, divorced or widowed)	24	31.79 ± 6.81			
Family income			5.06	0.006	0.083
Low	39	28.54 ± 6.75			
Medium	57	31.98 ± 5.32			
High	26	33.15 ± 6.47			
Place of residence			0.46	0.643	0.091
Rural	37	31.44 ± 6.30			
Urban	85	30.84 ± 7.06			
Religion			−0.54	0.59	0.247
Yes	117	31.19 ± 6.48			
No	5	32.80 ± 7.98			
Barthel index			6.72	0.002	0.102
>70	32	34.19 ± 5.63			
50 ~ 70	43	30 ± 7.56			
<50	47	28.81 ± 6.02			

A total of 122 participants completed the questionnaire in this study. Among them, 56.56% were male; 18.85% were under 50 years old; 32.79% had an educational attainment of junior high school or below; and 80.33% were married. Other demographic characteristics of the participants are shown in Table 2. The results showed that the comparison of Herth Hope Scale scores of stroke patients with different genders, educational levels, family income levels, and Barthel indexes showed statistically significant differences (*p* < 0.01) (see Table 2).

### 3.3 Correlation analysis between the level of social participation and the level of hope of stroke patients

Spearman correlation analysis revealed that social participation in stroke patients showed significant negative correlations with the total Hope Scale score and all subscale dimensions (*p* < 0.05). Scores for taking proactive actions and maintaining a positive attitude towards reality and future exhibited significant negative correlations with all four dimensions of the IPA (*p* < 0.05). Maintaining close relationships with others showed significant negative correlations with all other IPA dimensions except the outdoor independent participation dimension, as shown in Table 3.

**Table 3:** Correlation analysis between social participation and level of hope in 122 stroke patients (*r*-value).

Item	Taking positive action	Positive attitude toward reality and future	Maintaining close relationship with others	Hope
Indoor autonomous participation	−0.242**	−0.238**	−0.289**	−0.340**
Outdoor autonomous participation	−0.272**	−0.265**	−0.133	−0.298**
Autonomous participation in family roles	−0.269**	−0.263**	−0.432**	−0.422**
Autonomous participation in social life	−0.544**	−0.370**	−0.288**	−0.531**
Social participation	−0.445**	−0.377**	−0.401**	−0.539**

**P < 0.01.

### 3.4 Multiple linear regression analysis of the influence of general information and social participation level of stroke patients on their hope level

Multiple linear regression analysis was carried out with the variables with statistically significant differences in the univariate analysis and the social participation level as the independent variables, and with the total score of the Herth Hope Scale of patients as the dependent variable, and the method of assigning the values of the independent variables is shown in Table 4.

**Table 4:** Method for assigning independent variables in multiple linear regression analysis.

Independent variable	Assignment method
Gender	Male = 1, female = 2
Educational level	Junior high school and below = 1; high school or junior college = 2; college and above = 3
Family income level	Low = 1, medium = 2, high = 3
Barthel Index (points)	>70 = 1, 50–70 = 2, <50 = 3
Level of social participation	Original value entry

Multicollinearity tests revealed that the variance inflation factor values for all included variables were below 10.0, confirming the absence of multicollinearity among variables. The Durbin-Watson statistic was 1.798, falling within the acceptable range of 0–4 and indicating independent data. Furthermore, residual analysis demonstrated that residuals followed a normal distribution. The results of the multiple linear regression analysis revealed that the Barthel Index and social participation were significant predictors of stroke patients’ desired level of functioning (*p* < 0.05) after inclusion in the regression equation, explaining 47.4% of the total variance. This finding indicates that the Barthel Index and social participation are key factors influencing stroke patients’ desired level of functioning (see Table 5 for details).

**Table 5:** Multiple regression analysis of correlate factors for stroke patients.

Variables	*B*	*SE*	*β*	*t*	*P*	95% CI	*VIF*
Constant term	27.889	2.596	-	10.741	<0.001	22.746 ~ 33.031	
Gender	−0.211	0.89	−0.017	−0.237	0.813	−1.973 ~ 1.551	1.125
Educational level	0.264	0.895	0.024	0.295	0.769	−1.509 ~ 2.036	1.441
Household income level	1.075	0.687	0.126	1.564	0.121	−0.286 ~ 2.436	1.419
Barthel Index	0.102	0.02	0.382	5.022	<0.001	0.062 ~ 0.143	1.275
Social participation level	−0.15	0.028	−0.394	−5.422	<0.001	−0.205 ~ −0.095	1.163

*R* = 0.688, *R*^2^ = 0.474, adjusted *R*^2^ = 0.451, *F* = 20.869, *P*<0.001, “-”means no data. *P*-value: The *p*-value indicates whether the observed relationship is statistically significant. A *p* < 0.05 is considered statistically significant, meaning that this relationship is unlikely to have occurred by chance.

## 4 Discussion

### 4.1 Current status of social participation of stroke patients

In this study, stroke patients’ total social participation score was 32.60 ± 16.32, indicating a relatively low level in real-world settings, suggesting that stroke patients generally exhibit good social participation and autonomy. Across various dimensions, patients demonstrated strong performance in indoor activities, outdoor activities, autonomy in social life, and autonomy in general household roles. Multiple studies indicate that advances in healthcare and rehabilitation technologies have significantly increased opportunities for stroke patients to engage in diverse indoor and outdoor activities. VR-based rehabilitation therapy enables stroke patients to engage in realistic scenarios within a safe environment, thereby enhancing motivation, compliance, and neuroplasticity. This approach facilitates improvements in patients’ ability to perform daily tasks both indoors and outdoors (Aderinto et al., 2023). Furthermore, the implementation of personalized rehabilitation plans can enhance functional status, balance, mobility, and walking ability post-stroke, increasing participation in both indoor and outdoor activities (Langhammer and Lindmark, 2012). Reduced family role participation may reflect overprotection by caregivers and decreased household activity involvement (Šaňák et al., 2024). Conversely, patients received heightened social support post-stroke, leaving social relationships less affected (Deb-Chatterji et al., 2022), explaining the higher social autonomy scores. Prior research (Gurková et al., 2025) indicates that enhanced social participation improves quality of life.

### 4.2 Current status of hope levels in stroke patients

Hope represents a fundamental psychological construct that persists across the lifespan, reflecting an individual’s goal-directed expectations and motivations (Wu et al., 2025). The results of this study showed that the total score of hope level of stroke patients was (30.81 ± 7.28), which was above the medium level, and the hope level of stroke patients was affected by patients’ genders, educational levels, family income levels, and ability to perform activities of daily living (*p <* 0.05) similar to the results of Boru Sun et al. (2024). Our research indicates that men exhibit slightly higher levels of hope than women. Across diverse cultural contexts, women are often assigned caregiving roles, reflected in their traditional responsibilities for nurturing and household affairs. This transition from “caregiver” to “care recipient” coupled with perceptions of difficulty fulfilling family and parental obligations, leads women to experience more severe anxiety (Röding et al., 2003). This anxiety is significantly correlated with their levels of hope (Wang et al., 2019), thereby diminishing their sense of hope. This finding is inconsistent with the results of Labrosciano et al. (2025), possibly because our study sample size was too small to accurately represent the overall level of hope among stroke patients. Additionally, Matérne et al. (2025) found that men face greater difficulties in adapting after stroke and experience lower quality of life, which is inconsistent with our findings. One possible reason is that this study only included individuals aged 40–64 in its quantitative analysis, primarily focusing on middle-aged stroke patients. On the other hand, it may be because that study (*n* = 51) shares the same limitation as our study—a sample size too small to adequately represent the overall stroke patient population.

There is a positive correlation between patients’ literacy level and hope level, which may be because patients with a high literacy level have stronger cognition and coping ability to better adjust their mentality and face the disease positively. Patients with good family economic conditions have higher levels of hope. Good economic conditions can provide patients with better medical resources and life protection, reduce the economic and mental burden of patients, and thus raise the level of hope.

Furthermore, the findings of this study indicate that activities of daily living (ADL) capacity can significantly influence the level of hope among stroke patients. This aligns with the results reported by Fong et al. (2022). This may be attributed to the fact that improved ADL capacity fosters patients’ sense of autonomy and self-efficacy, both of which are key contributors to hope (Cheng et al., 2025). Consequently, patients with stronger ADL abilities tend to exhibit higher levels of hope. These results highlight vulnerable patient subgroups requiring targeted psychosocial interventions: older adults, those with limited education, low-income individuals, and patients with severe ADL impairment. Multidisciplinary approaches incorporating cognitive-behavioral strategies, family counseling, and community support programs may effectively enhance hope in these populations.

### 4.3 Analysis of the correlation between the level of social participation and the level of hope in stroke patients

The results of this study showed that the level of social participation of stroke patients was negatively correlated with the total score and the scores of each dimension of the Herth Hope Scale (*p <* 0.05). The results of multiple linear regression analysis showed that the level of social participation can positively affect the level of hope of patients. Social participation reflects patients’ perceptions of participation positivity, controlling and realizing their potential, and feeling the difficulties of social participation, which not only focuses on the satisfaction status of individual needs, but also attaches importance to the embodiment of personal social value (Zhou et al., 2022). It has been shown that social participation is conducive to enhancing psychological health (Douglas et al., 2017; Lin and Ren, 2021), life satisfaction, and wellbeing (Sun and Lyu, 2020). Social participation can divert patients’ attention from the role of “patient” and alleviate anxiety and depression (Chen et al., 2022); interaction with family, friends, and society can provide different kinds of resource exchange; work participation can significantly improve patients’ self-esteem and stabilize their emotions, which is important for maintaining positive attitudes and improving life satisfaction (Quan et al., 2016).

Studies have shown (Ge et al., 2021) that implementing interventions for stroke patients can increase the level of hope and adherence to functional exercise, improve neurological function, and enhance the ability to perform activities of daily living. The higher the level of social participation, the easier it is for patients to adapt to the change of social and family roles after the disease, the faster they can adjust their own psychological state (Zhao et al., 2020), and strive to seek medical and family support and help, actively cooperate with clinical treatment and rehabilitation training, and improve their own self-care ability, which is conducive to the improvement of the level of hope. On the contrary, patients with lower levels of social participation, facing different degrees of physical dysfunction, complications, and heavy medical burden, are prone to negative emotions such as low self-esteem and avoidance, so that the level of hope decreases (Mai et al., 2022).

Studies have shown (Zuo et al., 2023) that individuals with a high level of social engagement are more likely to have a desirable outcome when facing external challenges. Healthcare professionals should prioritize assessing the social participation levels of stroke patients and implement targeted cognitive-behavioral interventions. Key objectives include addressing patients’ low self-esteem, mitigating their lack of confidence in rehabilitation, and alleviating concerns about long-term prognosis. By guiding patients to develop accurate perceptions of their health status and fostering realistic expectations about recovery, clinicians can enhance patients’ self-efficacy in disease management. This approach cultivates a resilient, proactive mindset, ultimately elevating hope levels and optimizing health outcomes in stroke survivors.

The hope level among stroke patients remains suboptimal, particularly in females, individuals with lower educational attainment, limited economic income, and impaired activities of daily living. Targeted interventions are needed to enhance patients’ social participation, thereby fostering hope. During rehabilitation, a dual focus on physical recovery and psychological reconstruction is essential. Additionally, modifying patients’ living environments and cultivating supportive social contexts can improve outcomes. Comprehensive assessment and utilization of social support systems are critical to increasing participation rates and perceived engagement, ultimately promoting greater wellbeing.

## 5 Limitations of the study

First, the cross-sectional design limits the ability to establish a causal relationship between social participation and hope levels. Future longitudinal or mixed-methods studies are needed to determine causal pathways. Secondly, our study’s reliance on univariate significance for variable selection may overlook potential factors, leading to estimation bias. For instance, factors such as age, disease duration, stroke type, and comorbidities may also influence patients’ levels of hope. Future research should theoretically define covariates and construct models to comprehensively assess stroke patients’ levels of hope. Third, the study relied on self-reported questionnaires, which may introduce response bias. Participants’ answers may not accurately reflect actual circumstances. Future studies could incorporate multidimensional hope assessments (e.g., Herth Hope Index, Snyder Hope Scale) to enhance construct validity. Finally, this study employed convenience sampling with a small sample size from a single institution, limiting the generalizability of findings. Future large-scale, multicenter studies are needed to ensure the reliability of research outcomes.

## 6 Conclusion

This study highlights the critical role of enhancing social participation and hope levels among stroke patients. Findings indicate that both social participation and hope levels among stroke patients are moderate, with a positive correlation between the two. This provides new directions for developing interventions aimed at elevating hope levels in stroke patients.

Additionally, the findings reveal that individual characteristics—such as gender, educational attainment, household income, and activities of daily living (ADL) ability—influence hope levels among stroke patients. Future interventions should therefore be tailored to address these specific characteristics, thereby effectively elevating hope levels in this population.

## Data Availability

The original contributions presented in the study are included in the article/supplementary material, further inquiries can be directed to the corresponding author.
